# Incidence of Severe Neutropenia in HIV-Infected People Starting Antiretroviral Therapy in West Africa

**DOI:** 10.1371/journal.pone.0170753

**Published:** 2017-01-25

**Authors:** Charline Leroi, Eric Balestre, Eugene Messou, Albert Minga, Adrien Sawadogo, Joseph Drabo, Moussa Maiga, Marcel Zannou, Moussa Seydi, Francois Dabis, Antoine Jaquet, for the IeDEA West Africa collaboration

**Affiliations:** 1 Bordeaux University, School of Public Health (ISPED), Centre INSERM U1219-Epidemiologie-Biostatistique, Bordeaux, France; 2 INSERM, ISPED, Centre INSERM U1219-Epidemiologie-Biostatistique, Bordeaux, France; 3 Programme PAC-CI, CHU de Treichville, Abidjan, Côte d’Ivoire; 4 Centre de Prise en charge de Recherche et de Formation, Hôpital Yopougon Attié, Abidjan, Côte d’Ivoire; 5 Centre Médical de Suivi de Donneurs de Sang /CNTS/PRIMO-CI, Abidjan, Cote d’Ivoire; 6 Département de maladies infectieuses, CHU Souro Sanou, Bobo-Dioulasso, Burkina Faso; 7 Service de Médecine interne, CHU Yalgado Ouedraogo (CHU-YO), Ouagadougou, Burkina Faso; 8 Service d’Hépato-Gastro-Entérologie, Hôpital Gabriel Touré, Bamako, Mali; 9 Centre de Prise en Charge des Personnes vivant avec le VIH, CNHU, Cotonou, Benin; 10 Service de maladies infectieuses et tropicales, CRCF, CHU de Fann, Dakar, Sénégal; University of Pittsburgh Centre for Vaccine Research, UNITED STATES

## Abstract

**Background:**

In sub-Saharan Africa, antiretroviral therapy (ART) including drugs with potential toxicity such as Zidovudine (ZDV) are routinely prescribed. This study aimed at estimating the incidence of severe neutropenia and associated factors after ART initiation in five West African countries.

**Methods:**

A retrospective cohort analysis was conducted within the international epidemiologic database to evaluate AIDS (IeDEA) collaboration in West Africa. All HIV-infected adults, initiating ART between 2002 and 2014, with a baseline and at least one follow-up absolute neutrophil count (ANC) measurement were eligible. Incidence of severe neutropenia (ANC <750 cells/mm^3^) was estimated with 95% confidence interval (CI) according to age, gender, HIV clinic, hemoglobin, CD4 count, clinical stage, and ART duration. A Cox proportional hazard model was used to identify factors associated with severe neutropenia, expressed with their adjusted hazard ratios (aHR).

**Results:**

Between 2002 and 2014, 9,426 HIV-infected adults were enrolled. The crude incidence rate of a first severe neutropenia was 9.1 per 100 person-years (95% CI: 8.6–9.8). Factors associated with severe neutropenia were exposure to ZDV <6 months (aHR = 2.2; 95% CI: 1.8–2.6), ≥6–12 months (aHR = 2.1; 95% CI: 1.6–2.8) and ≥12 months (aHR = 1.6; 95% CI: 1.2–2.2) [Ref. no ZDV exposure], CD4 count <350 cells/mm^3^ (aHR = 1.3; 95% CI: 1.1–1.5) and advanced clinical stage at ART initiation (aHR = 1.2; 95% CI: 1.0–1.4).

**Conclusion:**

The incidence of severe neutropenia after ART initiation in West Africa is high and associated with ZDV exposure and advanced HIV disease. In this context, efforts are needed to scale-up access to less toxic first-line ART drugs and to promote early ART initiation.

## Introduction

Sub-Saharan Africa is by far the most affected region of the world by the HIV pandemic, with 25.8 million of people living with HIV (69.9% of the total) [1]. However, during the last decade, access to antiretroviral treatment (ART) has substantially improved in this part of the world and in 2014, 10.7 million of HIV-infected people (41.5% of the world total) were receiving ART in sub-Saharan Africa, an increase of 20% from 2010 [2]. These figures (absolute number and proportion) are likely to increase thanks to the recent World Health Organization (WHO) guidelines which recommends to initiate ART in all adults living with HIV regardless of CD4 cell count [3].

Facing an unprecedented number of people initiating ART in sub-Saharan Africa, the monitoring of treatment-related toxicity must be adapted to inform recommendations for the use of ART. Indeed, in low and middle-income countries long-term safety data of ART is still limited due to the lack of pharmacovigilance systems. Nevertheless, adverse events related to ART are common and represent the first cause of treatment modification in HIV-infected people on ART in sub-Saharan Africa [4,5]. The WHO classifies ART related toxicities into four grades of severity. In case of severe toxicity (grade 3–4) it is usually recommended to remove the incriminated drug. Mild or moderate neutropenia are usually asymptomatic but when severe, can lead to life-threatening infectious conditions and thus fit the grade 3–4 toxicity classification. Several studies have shown an increased risk of bacterial and fungal infections in HIV-infected people with neutropenia, most of them being conducted in the pre-ART era in industrialized countries [6–10]. In addition, a study has also revealed an increased risk of mortality associated with neutropenia in Spanish patients living with HIV [11].

Etiological factors leading to neutropenia in patients with HIV infection are multiple. Indeed, neutropenia can occur as a direct consequence of HIV infection and its related immunodeficiency, usually in the late stages of the HIV disease [12]. Another cause of neutropenia in people living with HIV is the use of myelotoxic agents. Despite the WHO 2013 guidelines recommending to treat everyone with tenofovir (TDF) in first-line [13], zidovudine (ZDV), one of the first-line antiretroviral drugs recommended since 2000, is still massively used.

There have been several studies reporting the impact of ZDV on developing neutropenia in a context of no or limited access to ART [14–17]. Other drugs such as cotrimoxazole (CTX), which is part of the minimal package of care for the prevention of opportunistic infection in people living with HIV, has also been shown to be a common cause of neutropenia [17–19].

In West Africa, where ZDV is still the usual drug in first-line ART regimens, there is a need to document the occurrence of hematologic toxicity in real-life conditions. The main objective of this study was to estimate the incidence of severe neutropenia and associated factors in people living with HIV initiating ART in West Africa.

## Methods

### Study population

The present analysis was conducted within the International Epidemiologic Databases to Evaluate AIDS (IeDEA) in West Africa consortium (www.iedea.org/) that aims at collecting a large set of data necessary for clinical and epidemiological research in the context of ART use [20]. This collaboration currently involves 17 HIV clinics from nine countries: Benin, Burkina Faso, Côte d’Ivoire, Ghana, Guinea-Bissau, Mali, Nigeria, Senegal and Togo. The IeDEA West Africa database maintains individual records related to socio-demographic, clinical and biological data of patients at ART initiation and during follow-up visits in participating health facilities. In each health center, patients are expected to have full blood count and selected biological tests at ART initiation and every six months thereafter. Data generated by these HIV clinics are reported, once a year, to the regional center based in Abidjan, Côte d’Ivoire using a standardized data format allowing data merging.

Adult patients (age ≥16 years) living with HIV and initiating a first-line ART regimen between 2002 and 2014 that contained three or more drugs, had an absolute neutrophil count (ANC) >750 cells/mm^3^ within six months prior to ART initiation and at least one follow-up ANC in the first six months on ART were included. HIV clinics not reporting ANC within their database were excluded from this project, leading a total of eight HIV clinics participating in this analysis: the Centre National Hospitalier Universitaire de Cotonou (CNHU) in Benin, the Centre Hospitalier Universitaire Souro Sanou of Bobo Dioulasso (BOBO) and the Service de Médecine interne du Centre Hospitalier Universitaire Yalgado Ouedraogo (CHUYO) in Burkina Faso, the Centre de Prise en charge de Recherche et de Formation de l’Hôpital Yopougon Attié (CePReF) of Abidjan, the Centre Médical de Suivi de Donneurs de Sang (CNTS) of Abidjan and the MTCT-Plus Program (MTCTP) of Abidjan in Côte d’Ivoire, the Service d’Hépato-Gastro-Entérologie du Centre Hospitalier Universitaire Yalgado Ouedraogo of Bamako (Gabriel Toure) in Mali and the Service de Maladies Infectieuses et Tropicales du Centre Hospitalier Universitaire de Fann in Dakar (SMITD) in Senegal.

### Data collection and outcome definition

For the present analysis, characteristics extracted from the IeDEA West Africa database were gender, date of birth, height, weight, ART regimen, clinical staging (either WHO or CDC definition), CD4 cell count and full blood count. Baseline was defined as the date of ART initiation.

The primary outcome of this study was severe neutropenia. Neutropenia was classified according to the NIH Division of AIDS scale: grade 1 (ANC <1,300 cells/mm^3^), grade 2 (ANC <1,000 cells/mm^3^), grade 3 (ANC <750 cells/mm^3^), and grade 4 (ANC <500 cells/mm^3^) [21]. Severe neutropenia was defined as an ANC ≤750 cells/mm^3^ (grade 3 or 4). The main exposure was ART and more specifically the use of ZDV. Exposure to ZDV was considered as a time-updated variable in order to take into account ART modifications over time. The cumulated exposure to ZDV was then classified into four categories: not exposed (reference group), exposed ≤6 months, exposed] 6–12 months] and exposed >12 months.

### Ethical considerations

The IeDEA West Africa Collaboration obtained authorization from the Ethics committee “Comité de Protection des Personnes Sud-Ouest et Outre-mer III” (N° 06–09) in Bordeaux, France, and from the national ethics committees of each participating countries: Benin (IRB 00006860), Burkina Faso (N° 2011-8-46), Cote d’Ivoire (IRB 00009111), Senegal (IRB 00002659) and Mali (IRB 00001465). A waiver for individual informed consent was given by the Institutional Review Boards as the data was collected from routine patients' charts. The study procedure did not involve any personal contact with the patients.

### Statistical analysis

The baseline characteristics were summarized using descriptive statistics. Missing values at baseline were imputed by the most updated data within a range of three months and data still missing were treated as a specific category. Longitudinal data from patients with a baseline ANC measure and at least one ANC measure after six month were computed to estimate the incidence rate of severe neutropenia and evaluate the factors associated with the occurrence of neutropenia. The incidence rate was defined as the number of patients with a first episode of severe neutropenia per 100 person-years (PY) of follow-up. Poisson regression method was used to estimate the incidence rate of neutropenia with its 95% confidence interval (CI). Incidence rates were adjusted for HIV clinic, gender, age, calendar year for ART initiation, baseline neutrophil count, baseline hemoglobin level, baseline CD4 count, first-line ART regimen and baseline clinical stage.

The role of ART and other characteristics in the development of first-time neutropenia was analyzed by univariate and multivariate Cox proportional hazards regression models. All the explanatory variables were considered at baseline except ZDV exposure and CD4 count that were computed as time-updated variables. In order to take into account time-updated variables we did a counting process format style of input which consists of a sequence of time intervals over which the values of any time-varying explanatory variable are constant for each participating individual. The final multivariate Cox regression model was stratified on HIV clinic and only significant variables (p <0.05) and possible confounding factors were kept in the model. Each patient contributed to the time at risk from the time they entered into the study (i.e. the date of ART initiation) either until the closing date (December 31^st^, 2014), date of death or loss-to-follow-up, date of transfer out, date of last visit with ANC available in an interval of six months or date of first severe neutropenia. We restricted the analysis for a two-year period after ART initiation. In addition, we described the ART modifications that occurred in the month following the development of severe neutropenia.

All statistical analysis was performed with the SAS 9.4 software (SAS Institute Inc., Cary, NC, USA).

## Results

**Fig 1:**
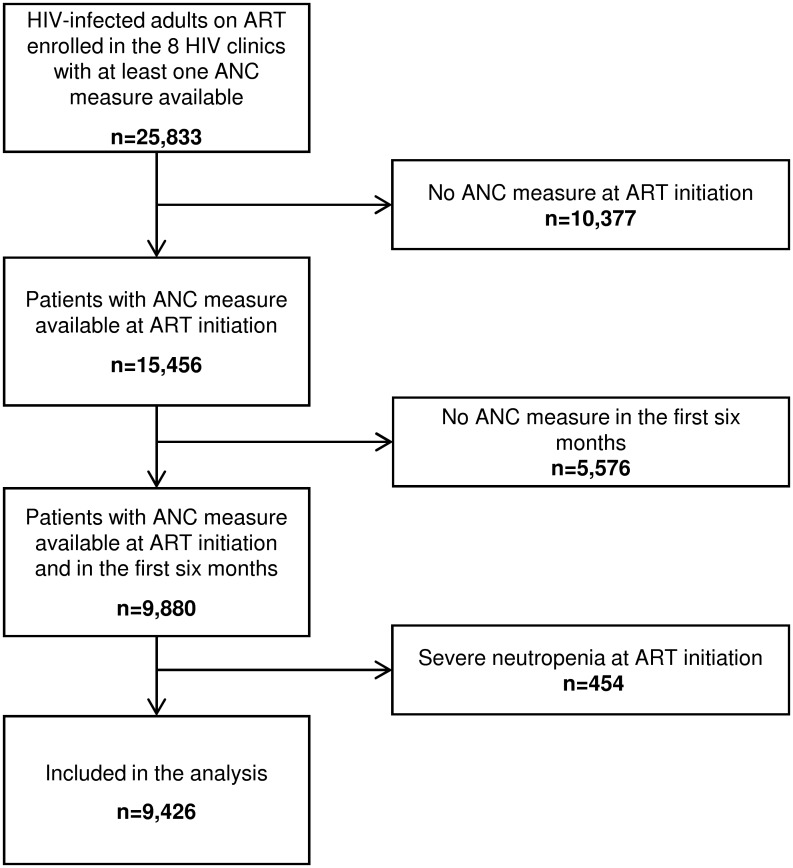
Study population flow chart. ANC, absolute neutrophil count; ART, antiretroviral therapy.

Of the 25,833 patients with at least one ANC measure available enrolled in the participating IeDEA West Africa cohorts between 2002 and 2014, 15,456 (60%) had an ANC measure available at baseline (ART initiation). Of these, 9,880 (65%) had at least another ANC during the first six months but 454 of them were excluded because of severe neutropenia at ART initiation. Overall, 9,426 patients were included in this analysis (Fig 1).

Patients with at least one ANC measure available but who were excluded (n = 16,407) initiated ART in earlier calendar year than the 9,426 patients included in the analysis (median year: 2008 versus 2009; p <10^−4^). Conversely, there were no significant differences concerning age (p = 0.11), gender (p = 0.20), baseline CD4 count (p = 0.18) and ART regimen distribution (p = 0.49) (S1 Table).

Baseline characteristics of the included patients are shown in Table 1; 70% were women and the median age was 37 years [Inter-quartile Range (IQR): 31–44]. The median calendar year for ART initiation was 2009 [IQR: 2006–2011]. The median CD4 count at ART initiation was 174 cells/mm^3^ [IQR: 80–278]. The proportion of patient with either CDC stage 3 or WHO stage 4 was 16.5%. Grade 1 neutropenia was reported in 11% at ART initiation and grade 2 neutropenia in 5%. The median hemoglobin level was 10g/dL [IQR: 9–12]. Moderate and severe anemia was found in 25% and 7% of included patients, respectively. A ZDV-containing ART regimen was initiated in 5,081 (54%) patients.

**Table 1:** Patient’s characteristics at ART initiation. IeDEA West Africa collaboration, 2002–2015 (N = 9,426).

Characteristics	All (N = 9,426)	
**HIV clinic, Country; n (%)**		
CePReF, Cote d’Ivoire	3,529	(37.4)
CNTS, Cote d’Ivoire	1,502	(15.9)
MTCTP, Cote d’Ivoire	462	(4.9)
BOBO, Burkina Faso	1,956	(20.8)
CHUYO, Burkina Faso	279	(3.0)
CNHU, Benin	816	(8.7)
Gabriel Toure, Mali	559	(5.9)
SMITD, Senegal	323	(3.4)
**Calendar year for ART initiation; median [IQR]**	2009	[2006–2011]
**Gender; n (%)**		
Female	6,598	(70.0)
Male	2,828	(30.0)
**Age (years); n (%)**		
<35	4,024	(42.7)
≥35	5,402	(57.3)
**Clinical stage (CDC 3 or WHO 4); n (%)**		
No	5,930	(62.9)
Yes	1,557	(16.5)
Missing	1,939	(20.6)
**CD4 count (cells/mm**^**3**^**); n (%)**		
<350	7,314	(77.6)
≥350	1,122	(11.9)
Missing	990	(10.5)
**Neutrophils count (cells/mm**^**3**^**); n (%)**		
≥1,300	7,944	(84.3)
1,000–1,300	1,034	(11.0)
750–1,000	448	(4.8)
**Hemoglobin level (g/dL); n (%)**		
≥10	5,974	(63.4)
7.5–10	2,307	(24.5)
<7.5	629	(6.7)
Missing	516	(5.5)
**ART regimen containing ZDV; n (%)**		
No	4,345	(46.1)
Yes	5,081	(53.9)
**First-line ART regimen; n (%)**		
ZDV/3TC/EFV	2,195	(23.3)
ZDV/3TC/NVP	2,307	(24.5)
D4T/3TC/EFV	819	(8.7)
D4T/3TC/NVP	1,426	(15.1)
TDF-based regimens	1,590	(16.9)
Other regimens	1,089	(11.6)

IQR, interquartile range; CI, confidence interval; ART, antiretroviral therapy; CDC, US Centers for Disease Control; WHO, World Health Organization; ZDV, zidovudine; 3TC, lamivudine; EFV, efavirenz; D4T, stavudine; NVP, nevirapine; TDF, tenofovir; FTC, emtricitabine.

**Fig 2:**
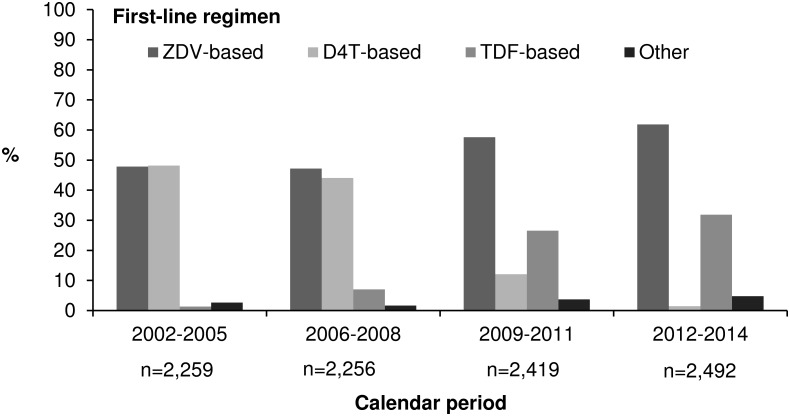
Distribution of first-line antiretroviral regimens prescribed according to calendar period. **IeDEA West Africa collaboration, 2002–2014 (N = 9,426)**. ZDV, zidovudine; D4T, stavudine; NVP, nevirapine; TDF, tenofovir.

As depicted in Fig 2, the proportion of patients initiating a D4T-containing regimen in our study population fell from 50% between 2002 and 2008 to 12% during the period 2009–2011 and to only 1% for the period 2012–2014. This decreased was compensated by an increased prescription of ZDV and tenofovir (TDF)-containing regimens. Indeed, before 2009, ZDV was prescribed just as much as D4T and its proportion increased to >60% for the period 2012–2014 and remained the most frequently prescribed combination since then. The proportion of patients initiating a TDF-containing regimen increased from <8% before 2009 to 27% for the period 2009–2011 and 32% for the period 2012–2014.

### Incidence of severe neutropenia

From 2002 to 2014, the 9,426 patients enrolled contributed to 9,724 person-years of follow-up. The median participation time was 12 months [IQR: 6–20] during which 888 patients presented at least one episode of severe neutropenia. The overall incidence rate of severe neutropenia was estimated at 9.1 per 100 PY (95% CI: 8.6–9.8) (Table 2). This estimate was significantly different between HIV clinics (p <10^−4^), from 3.5 per 100 PY in BOBO, Burkina Faso to 13.0 in CePReF, Cote d’Ivoire. The incidence was also significantly higher in patients exposed to an ART regimen containing ZDV (p <10^−4^), starting ART with a CD4 count <350 cells/mm^3^ (p <10^−4^), at clinical stage CDC 3 or WHO 4 (p <10^−3^) and in patients with neutropenia of grade 1 or 2 (p <10^−4^). The neutropenia rate in patients who initiated ART after 2005 was lower than in patients who initiated ART between 2002 and 2005. The occurrence of severe neutropenia did not differ by gender, age and baseline hemoglobin level.

**Table 2:** Incidence of severe neutropenia among 9,426 HIV-infected patients over a two-year period after ART initiation. IeDEA West Africa collaboration, 2002–2015.

Characteristic	No. patients	No. of first neutropenia / PY	Rate (95% CI) per 100 PY	p
**HIV clinic, Country**				<10^−4^
CePReF, Cote d’Ivoire	3,529	481/3,701	13.0 (11.9–14.2)	
CNTS, Cote d’Ivoire	1,502	141/1,766	8.0 (6.8–9.4)	
MTCTP, Cote d’Ivoire	462	50/386	13.0 (9.8–17.1)	
BOBO, Burkina Faso	1,956	93/2,638	3.5 (2.9–4.3)	
CHUYO, Burkina Faso	279	18/155	11.7 (7.3–18.5)	
CNHU, Benin	816	46/446	10.3 (7.7–13.8)	
Gabriel Toure, Mali	559	33/405	8.1 (5.8–11.5)	
SMITD, Senegal	323	26/228	11.4 (7.8–16.8)	
**Gender**				0.50
Male	2,828	265/2,828	9.5 (8.4–10.7)	
Female	6,598	623/6,923	9.0 (8.0–9.7)	
**Age (years)**				0.39
<35	4,024	364/4,123	8.8 (8.0–9.8)	
≥35	5,402	524/5,601	9.3 (8.6–10.2)	
**Calendar year for ART initiation**				<10^−4^
2002–2005	2,259	327/2,270	14.4 (12.9–16.1)	
2006–2008	2,256	193/2,485	7.8 (5.8–10.3)	
2009–2011	2,419	190/2,860	6.6 (5.0–8.8)	
2012–2014	2,492	178/2,110	8.4 (6.3–11.3)	
**Baseline neutrophils count (cells/mm**^**3**^**)**				<10^−4^
≥1,300	7,944	649/8,238	7.9 (7.3–8.5)	
1,000–1,300	1,034	145/1,064	13.6 (11.6–16.0)	
750–1,000	448	94/422	22.3 (18.2–27.3)	
**Baseline hemoglobin level (g/dL)**				0.56
≥10	5,974	572/6,383	9.0 (8.3–9.7)	
<10	2,936	275/2,954	9.3 (8.3–10.5)	
Missing	516	41/387	10.6 (7.8–14.4)	
**Baseline CD4 count (cells/mm**^**3**^**)**				<10^−4^
≥350	1,122	56/1,272	4.4 (3.4–5.8)	
<350	7,314	753/7,512	10.0 (9.3–10.8)	
Missing	990	79/941	3.7 (2.6–5.2)	
**Initial ART regimen containing ZDV**				<10^−4^
No	4,345	333/4,353	7.7 (6.9–8.5)	
Yes	5,081	555/5,372	10.3 (9.5–11.2)	
**First-line ART regimen**				<10^−4^
ZDV/3TC/EFV	2,195	264/2,281	11.6 (10.3–13.1)	
ZDV/3TC/NVP	2,307	240/2,433	9.9 (8.7–11.2)	
D4T/3TC/EFV	819	86/833	10.3 (8.4–12.8)	
D4T/3TC/NVP	1,426	127/1,446	8.8 (7.4–10.5)	
TDF-based regimens	1,590	85/1,542	5.5 (4.5–6.8)	
Other regimens	1,089	86/1,189	7.2 (5.9–8.9)	
**Baseline clinical stage (CDC 3 or WHO 4)**				<10^−3^
No	5,930	527/6,184	8.5 (7.8–9.3)	
Yes	1,557	184/1,552	11.9 (10.3–13.7)	
Missing	1,939	177/1,988	8.9 (7.7–10.3)	

PY, person-year; CI, confidence interval; ART, antiretroviral therapy; CDC, US Centers for Disease Control; WHO, World Health Organization; ZDV, zidovudine; 3TC, lamivudine; EFV, efavirenz; D4T, stavudine; NVP, nevirapine; TDF, tenofovir; FTC, emtricitabine; CTX, cotrimoxazole.

### Factors associated with severe neutropenia

The results of Cox regression models are shown in Table 3. The risk to develop a first episode of severe neutropenia was significantly higher in patient exposed to ZDV compared to those initiating another first-line ART regimen, irrespective of their time spent on ZDV:] 0–6] months (adjusted hazard ratio (aHR) = 2.15; 95% CI: 1.81–2.56),] 6–12] months (aHR = 2.09; 95% CI: 1.60–2.72) and >12 months (aHR = 1.62; 95% CI: 1.18–2.22) (Table 3). The presence of a mild or moderate neutropenia at baseline was associated with an increased risk of subsequent episode of severe neutropenia. Other factors associated with the occurrence of severe neutropenia were: initiating ART between 2009 and 2011 (aHR = 0.73; 95% CI: 0.60–0.91) or between 2012 and 2014 (aHR = 0.55; 95% CI: 0.44–0.69) compared to initiation before 2005; an advanced clinical stage (CDC 3 or WHO 4) at ART initiation (aHR = 1.21; 95% CI: 1.02–1.44); and time-updated CD4 count <350 cells/mm^3^ (aHR = 1.26; 95% CI: 1.07–1.49) compared with CD4 count ≥350cells/mm^3^.

**Table 3:** Cox regression univariate and multivariate models estimating risk of severe neutropenia within two years after ART initiation. IeDEA West Africa collaboration, 2002–2014 (N = 9,426).

Characteristic	Univariate analysis			Multivariate analysis^1^		
	HR	95% CI	p	aHR	95% CI	p
**Time-updated exposure to ZDV (months)**			<10^−4^			<10^−4^
0 (Not exposed)	1	Ref.		1	Ref.	
]0–6]	1.80	1.52–2.14		2.15	1.81–2.56	
]6–12]	1.81	1.39–2.35		2.09	1.60–2.72	
>12	1.37	1.01–1.86		1.62	1.18–2.22	
**Calendar year for ART initiation**			<10^−4^			<10^−4^
2002–2005	1	Ref.		1	Ref.	
2006–2008	0.56	0.47–0.66		0.87	0.71–1.06	
2009–2011	0.49	0.41–0.59		0.73	0.60–0.91	
2012–2014	0.56	0.46–0.67		0.55	0.44–0.69	
**Baseline neutrophils count (cells/mm**^**3**^**)**			<10^−4^			<10^−4^
≥1,300	1	Ref.		1	Ref.	
1,000–1,300	1.72	1.44–2.06		1.52	1.27–1.82	
750–1,000	2.74	2.20–3.40		2.47	1.99–3.07	
**Baseline hemoglobin level (g/dL)**			0.86			0.25
≥10	1	Ref.		1	Ref.	
<10	1.03	0.89–1.19		1.13	0.97–1.32	
Missing	1.07	0.78–1.47		0.90	0.55–1.47	
**Clinical stage (CDC 3 or WHO 4) at initiation**			<10^−4^			<0.01
No	1	Ref.		1	Ref.	
Yes	1.39	1.17–1.64		1.21	1.02–1.44	
Missing	1.04	0.88–1.24		1.59	1.08–2.34	
**Time-updated CD4 count (cells/mm**^**3**^**)**			<10^−4^			<0.01
≥350	1	Ref.		1	Ref.	
<350	1.41	1.19–1.66		1.26	1.07–1.49	
Missing	1.11	0.85–1.46		1.57	1.11–2.22	

^1^Analyses were stratified on HIV clinics.

HR, hazard ratio; aHR, adjusted hazard ratio; CI, confidence interval; ZDV, zidovudine; CDC, US Centers for Disease Control; WHO, World Health Organization.

Thereafter, we looked at ART modification after a first episode of severe neutropenia (Table 4). Of the 888 patients with severe neutropenia, 128 (14.4%) had a change of ART regimen in the following month corresponding to a total of 134 drug modifications. The first drug stopped was ZDV (49%) followed by Nevirapine (24%). Among the 65 patients who stopped ZDV after severe neutropenia, 43% switched to D4T and 46% to TDF.

**Table 4:** Distribution of antiretroviral modifications in HIV-infected patients with a first severe episode of neutropenia (n = 134).

Drug stopped	Distribution of modifications						Total
	ZDV	D4T	TDF	EFV	NVP	Other	
ZDV	-	28 (43%)	30 (46%)	0 (0%)	0 (0%)	7 (11%)	65
D4T	11 (61%)	-	2 (11%)	0 (0%)	0 (0%)	5 (28%)	18
TDF	5 (71%)	2 (29%)	-	0 (0%)	0 (0%)	0 (0%)	7
EFV	0 (0%)	0 (0%)	0 (0%)	-	8 (67%)	4 (33%)	12
NVP	0 (0%)	0 (0%)	0 (0%)	26 (81%)	-	6 (19%)	32
**Total**	**16 (12%)**	**30 (22%)**	**32 (24%)**	**0 (0%)**	**0 (0%)**	**0 (0%)**	**134**

ZDV, zidovudine; 3TC, lamivudine; EFV, efavirenz; D4T, stavudine; NVP, nevirapine; TDF, tenofovir.

## Discussion

We found that the risk of developing severe neutropenia was associated with ZDV-containing ART regimen, initiating ART before 2009, having a low baseline neutrophil count, an advanced clinical stage and a more profound immune deficiency. In addition we found that the risk of severe neutropenia persisted over six months of treatment with ZDV-containing ART regimens in a context where the majority of ART initiation were based on ZDV including in the recent years, despite the change in WHO recommendations to replace it by TDF [13].

Our estimated incidence of severe neutropenia was higher than among patients living with HIV before access to ART in comparable settings. Indeed, the incidence rate of neutropenia reported by Toure *et al*. in a cohort study in Cote d’Ivoire was 4.3 per 100 PY after initiation of CTX prophylaxis [18]. Similarly, Anglaret *et al*. found an overall incidence rate of 1.9 per 100 PY during a CTX versus placebo clinical trial in Cote d’Ivoire and the incidence in the CTX arm was 2.8 per 100 PY [19]. Although theses variations might be related to methodological differences, they may also suggest that the incidence of severe neutropenia was lower in sub-Saharan Africa before access to ART. Conversely, our estimate is much lower than the rate of 56.3 per 100 PY found by Moh *et al*. in their cohort study in Cote d’Ivoire during the six months after ZDV-based ART initiation [22]. This variation can be explained by the fact that the majority of their study population (80%) was exposed to both ZDV and CTX prophylaxis at ART initiation, a combination known to increase the risk of severe neutropenia. As the impact of CTX on ANC is supposed to be acute, information on CTX use over time was needed to capture the impact of this molecule on neutropenia. Information on CTX exposure was incomplete in our study and we could not incorporate this variable in our analysis. Thus we could not fully compare our findings with the previous reports and their wide range of estimates of severe neutropenia.

We found an increased risk of severe neutropenia in patients exposed to ZDV and this risk was persistent after one year of treatment. These results are consistent with the known myelosuppressive effect of ZDV [14–17]. Hoffman *et al*. in South Africa reported an increased incidence of severe neutropenia after ZDV-based ART initiation in their cohort [17]. However, to the best of our knowledge, it is the first time that a study considers ZDV-exposure as a time-updated variable and quantifies the duration of exposure.

We also found that advanced immunodeficiency and advanced clinical stage were independent risk factors for severe neutropenia. These results are consistent with previous findings and emphasize the need of an early ART initiation as recently recommended by the WHO guidelines [3,14,18].

Finally, we found significant differences in the occurrence of severe neutropenia according to clinics. Several explanations may be formulated. The clinics participating to the IeDEA West Africa collaboration have their own organization and patient management procedures. Thus, the frequency of follow-up visits and biological measurements may vary substantially from one clinic to another, inducing a potential information bias. Second, unmeasured confounding factors associated with clinics might have influence the occurrence of severe neutropenia.

Despite the WHO recommendations suggesting to adapt the ART regimen when adverse effects of grade 3 or 4 occur, the rate of patients with severe neutropenia who changed ART regimen following neutropenia was low, leading to a few hypotheses. First, we were not able to document exposure to other drugs co-administered with ART such as CTX prophylaxis. CTX has a known acute hematologic toxicity and might be discontinued prior to any other drugs. If neutropenia resolves, CTX needs to be permanently evicted from patient’s prescriptions and no additional ART modifications are needed. Secondly, the limited availability of alternative ART regimens might have prevented any ART modification in the absence of clinical consequences associated with severe neutropenia. Thirdly, the reported ANC might have not been properly graded, thus limiting the applications of guidelines recommendations. Indeed, a previous report from our collaboration in West Africa showed that only a limited number of ART prescribers declared grading adverse drug reactions [4].

We acknowledge several limitations to our study. First, we selected patients with at least one ANC at baseline and one ANC during the first six months of follow-up. It might have introduced a bias of selection if patients included and excluded were different. However, we found no significant differences between these two groups concerning the CD4 count and exposure to ZDV at ART initiation. Second, the occurrence of neutropenia is expected in patients initiating a ZDV-based ART regimen leading to a potential bias of information if these patients were better monitored. However, we did not find any significant differences concerning the number of follow-up visits and the duration of follow-up between patients exposed to ZDV and patients not exposed. Another important limitation was the lack of information concerning the exposure to CTX. Without this information we were not able to describe the combined impact of exposure to ZDV and CTX. In addition, we could not take into account the CTX prophylaxis interruptions in our description of treatment modifications after severe neutropenia.

## Conclusion

The incidence of severe neutropenia was high among people living with HIV on ART in West Africa. Initiating ART with a ZDV-based regimen was an important risk factor associated with the occurrence of a severe neutropenia and this risk persisted over time. In addition, an advanced HIV disease and a low CD4 count at ART initiation were also associated with a subsequent risk to develop a severe neutropenia.

The recent WHO guidelines recommend initiating a TDF-based ART regimen in HIV-infected persons regardless of their CD4 count. In a context where ZDV is still massively prescribed and most patients initiate ART at an advanced stage of the disease with low CD4 count, the implementation of the WHO recommendation will be crucial in order to reduce the risk of severe neutropenia.

Severe neutropenia is a known risk factor for bacterial infections although we were not able to document this clinical outcome in our population followed for a relatively short period of time. Thereafter, a better documentation of morbidity is needed in order to assess the potential impact of drug therapies on clinical outcomes among HIV-infected persons in sub-Saharan Africa.

## Supporting Information

S1 Table Comparison of characteristics at ART initiation between included (N = 9,426) and excluded patients (N = 16,407). **IeDEA West Africa collaboration, 2002–2015**. IQR, interquartile range; ART, antiretroviral therapy; CDC, US Centers for Disease Control; WHO, World Health Organization; ZDV, zidovudine; 3TC, lamivudine; EFV, efavirenz; D4T, stavudine; NVP, nevirapine; TDF, tenofovir; FTC, emtricitabine.
(DOCX)
