# Supplementary material for: Incidence of Severe Neutropenia in HIV-Infected People Starting Antiretroviral Therapy in West Africa
Source: PLoS One. 2017 Jan 25;12(1):e0170753. doi: 10.1371/journal.pone.0170753 (PMC5266303; doi:10.1371/journal.pone.0170753)
Supplement: S1 Table — IeDEA West Africa collaboration, 2002–2015. IQR, interquartile range; ART, antiretroviral therapy; CDC, US Centers for Disease Control; WHO, World Health Organization; ZDV, zidovudine; 3TC, lamivudine; EFV, efavirenz; D4T, stavudine; NVP, nevirapine; TDF, tenofovir; FTC, emtricitabine. (DOCX) [file pone.0170753.s001.docx]

| **Characteristics** | | **Included patients** | |  | **Excluded patients** | | **p** |
| --- | --- | --- | --- | --- | --- | --- | --- |
|  |  |  |  |  |  |  |  |
|  |  | N=9,426 | |  | N=16,407 | |  |
| **Gender, n (%)** | |  |  |  |  |  | 0.20 |
| Female | | 2,828 | 30.0 |  | 5,047 | 30.8 |  |
| Male | | 6,598 | 70.0 |  | 11,360 | 69.2 |  |
| **Age (years), median [IQR]** | | 37 | [31-44] |  | 36 | [31-43] | 0.11 |
| **Calendar year for ART initiation; median [IQR]** | | 2009 | [2006-2012] |  | 2008 | [2006-2011] | <10^-4^ |
| **Clinical stage (CDC 3 or WHO 4); n (%)** | |  |  |  |  |  | <10^-4^ |
| No | | 5,813 | 61.7 |  | 9326 | 56.8 |  |
| Yes |  | 1,527 | 16.2 |  | 1853 | 11.3 |  |
| Missing | | 2,086 | 22.1 |  | 5228 | 31.9 |  |
| **CD4 count (cells/mm^3^); median [IQR]** | | 178 | [81-284] |  | 166 | [70-285] | 0.18 |
| **ART regimen containing ZDV; n (%)** | |  |  |  |  |  | 0.07 |
| No |  | 4,345 | 46.1 |  | 7754 | 47.3 |  |
| Yes |  | 5,081 | 53.9 |  | 8653 | 52.7 |  |
| **Initial ART combination, n (%)** | |  |  |  |  |  | 0.49 |
| ZDV/3TC/EFV | | 2,195 | 23.3 |  | 3835 | 23.4 |  |
| ZDV/3TC/NVP | | 2,307 | 24.5 |  | 3843 | 23.4 |  |
| D4T/3TC/EFV | | 819 | 8.7 |  | 1440 | 8.8 |  |
| D4T/3TC/NVP | | 1,426 | 15.1 |  | 2558 | 15.6 |  |
| TDF-based regimens | | 1,590 | 16.9 |  | 2775 | 16.9 |  |
| Other | | 1,089 | 11.6 |  | 1956 | 11.9 |  |
